# Takayasu arteritis with a giant ascending aortic pseudoaneurysm followed by a postoperative anastomotic pseudoaneurysm: a life-saving case report

**DOI:** 10.1093/ehjcr/ytag430

**Published:** 2026-06-09

**Authors:** Shota Inoue, Yukihiro Hayatsu, Yoshun Sai, Naoya Terao, Masaki Hata

**Affiliations:** Department of Cardiovascular Surgery, Sendai Kousei Hospital, 1-20 Tsutsumidori-Amamiyamachi, Aoba-ku, Sendai, Miyagi 981-0914, Japan; Department of Cardiovascular Surgery, Sendai Kousei Hospital, 1-20 Tsutsumidori-Amamiyamachi, Aoba-ku, Sendai, Miyagi 981-0914, Japan; Department of Cardiovascular Surgery, Sendai Kousei Hospital, 1-20 Tsutsumidori-Amamiyamachi, Aoba-ku, Sendai, Miyagi 981-0914, Japan; Department of Cardiovascular Surgery, Sendai Kousei Hospital, 1-20 Tsutsumidori-Amamiyamachi, Aoba-ku, Sendai, Miyagi 981-0914, Japan; Department of Cardiovascular Surgery, Sendai Kousei Hospital, 1-20 Tsutsumidori-Amamiyamachi, Aoba-ku, Sendai, Miyagi 981-0914, Japan

**Keywords:** Takayasu arteritis, Aortic pseudoaneurysm, Anastomotic pseudoaneurysm, Endovascular repair, Stent graft, Case report

## Abstract

**Background:**

Takayasu arteritis is a rare, chronic large-vessel vasculitis that predominantly affects young women, involving the aorta and its major branches. Pseudoaneurysm formation is uncommon in this disease, particularly in the ascending aorta, with only a few such cases having been reported.

**Case Summary:**

A 20-year-old Asian man presented with fever, cough, and haemoptysis. Contrast-enhanced computed tomography revealed a giant ascending aortic pseudoaneurysm (57 mm in diameter) compressing the pulmonary artery. Emergency total arch replacement was performed because the patient was in a pre-shock state and the risk of imminent rupture was high. Histopathology confirmed a diagnosis of Takayasu arteritis. The patient was discharged on postoperative Day 13 but returned 6 days later with a persistent low-grade fever, fatigue, and back pain. Repeat imaging revealed an anastomotic pseudoaneurysm between the left common carotid artery and the branch of the prosthetic graft. Emergency endovascular repair was performed using a stent graft. Corticosteroid therapy was initiated the following day, and the patient was transferred to the rheumatology department on postoperative Day 15 following the second intervention.

**Discussion:**

A giant pseudoaneurysm from Takayasu arteritis can cause pulmonary artery compression and acute haemodynamic compromise, requiring prompt surgical intervention. In such cases, adequate control of vascular inflammation may be important for reducing the risk of postoperative anastomotic complications. Endovascular stent grafting may serve as a viable treatment option for arch branch anastomotic pseudoaneurysms in the postoperative setting.

Learning points

- Ascending aortic pseudoaneurysm is a rare but potentially life-threatening complication of Takayasu arteritis that may compress the pulmonary artery and cause acute circulatory collapse.
- Endovascular stent grafting may represent an effective treatment for postoperative arch branch anastomotic pseudoaneurysms in Takayasu arteritis.
- Optimal perioperative control of vascular inflammation is essential in Takayasu arteritis to reduce the risk of postoperative complications following major aortic surgery.

## Introduction

Takayasu arteritis (TA), involving the aorta and its major branches, is a chronic large-vessel vasculitis that predominantly affects young women and is more prevalent in Asian populations.^1–3^ While vascular stenosis and aneurysmal dilatation represent common manifestations, pseudoaneurysm formation is rare, particularly in the ascending aorta.^4–6^

We report an unusual case of a young man with TA who developed a giant ascending aortic pseudoaneurysm leading to pulmonary artery compression and a pre-shock state. A postoperative arch branch anastomotic pseudoaneurysm subsequently developed and was successfully treated with endovascular stent grafting.

## Summary figure

**Figure:**
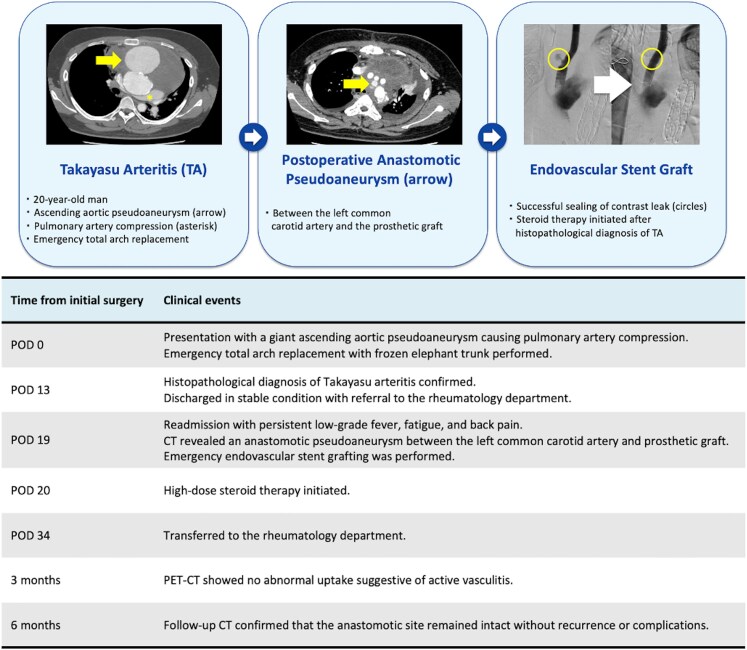
Clinical course of a 20-year-old man with TA presenting with a giant ascending aortic pseudoaneurysm compressing the pulmonary artery. Total arch replacement was performed, which was followed by the development of a postoperative anastomotic pseudoaneurysm between the left common carotid artery and the prosthetic graft. Endovascular stent grafting successfully sealed the lesion.

## Case presentation

A 20-year-old Asian man presented with a 1-week history of fever, cough, and haemoptysis. His medical and family history were unremarkable. On arrival, he was afebrile (36.8°C) with a blood pressure of 125/97 mmHg and heart rate of 122 bpm (shock index: 0.98). Physical examination revealed no cardiac murmurs. Laboratory tests showed leucocytosis (13 200/μL) and elevated C-reactive protein (CRP) level (16.89 mg/dL).

Transthoracic echocardiography revealed a hypoechoic mass compressing the pulmonary artery, accompanied by right ventricular dilation, mild tricuspid regurgitation [tricuspid regurgitant pressure gradient (TRPG): 30 mmHg], and dilation of the inferior vena cava (25 mm) without respiratory variation. Contrast-enhanced computed tomography (CT) demonstrated a giant ascending aortic pseudoaneurysm with a maximal diameter of 57 mm, associated with periaortic haematoma and disruption of aortic wall continuity, causing compression of the main pulmonary artery (*Figure 1*).

**Figure 1:**
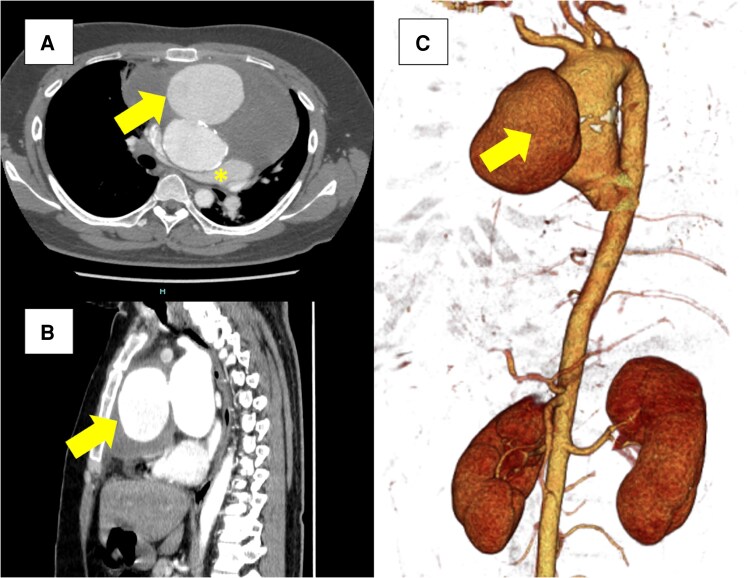
Preoperative contrast-enhanced computed tomography images. (*A*) Axial view showing a giant pseudoaneurysm (arrow) arising from the ascending aorta, with compression of the main pulmonary artery (asterisk). (*B*) Sagittal view showing an anteriorly protruding pseudoaneurysm. (*C*) Three-dimensional reconstructed image demonstrating the spatial relationship of the pseudoaneurysm.

This clinical condition was considered a pre-shock state due to pulmonary artery compression caused by the pseudoaneurysm, and an impending rupture could not be ruled out. Emergency total arch replacement with open stent grafting was performed. Intraoperatively, a 1.5 × 1.5-cm perforation was identified in the anterior wall of the ascending aorta. A 23 × 60-mm FROZENIX stent graft (Japan Lifeline Inc., Tokyo, Japan) was inserted through the open distal aortic stump, followed by total arch replacement using a four-branched 22 mm J-graft (Japan Lifeline Inc.; *Figure 2*). In this case, the frozen elephant trunk was used to achieve a secure distal anastomosis.

**Figure 2:**
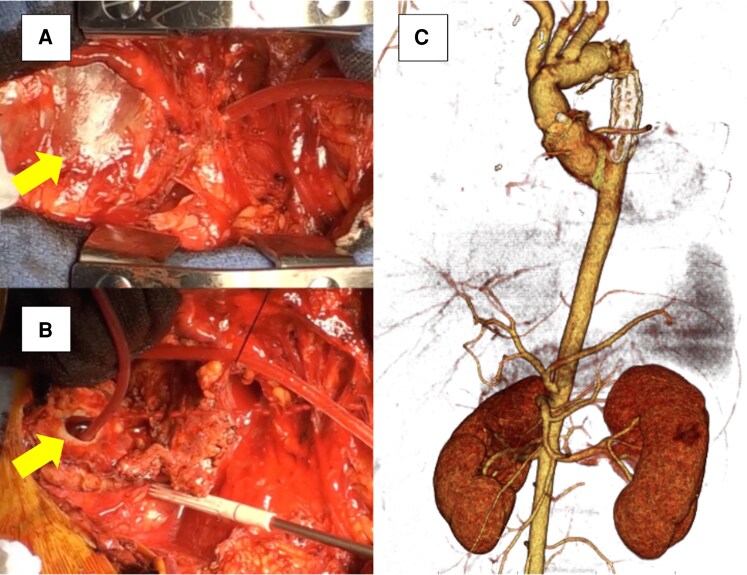
Intraoperative findings and postoperative contrast-enhanced computed tomography images. (*A*) Intraoperative image showing a giant pseudoaneurysm (arrow) arising from the ascending aorta. (*B*) Intraoperative image showing a 1.5 × 1.5-cm perforation (arrow) in the anterior wall of the ascending aorta. (*C*) Postoperative three-dimensional reconstructed image showing no abnormality at the anastomotic site.

Histopathological examination of the resected aortic wall revealed adventitial thickening and loss of medial elastic fibres. These characteristic findings confirmed a diagnosis of TA (*Figure 3*). Follow-up CT showed no abnormalities. Transthoracic echocardiography after relief of the pulmonary artery compression demonstrated improvement of right ventricular dilation and tricuspid regurgitation (TRPG: 17.2 mmHg), along with normalization of the inferior vena cava diameter (13 mm) and restoration of respiratory variation. The CRP level decreased to 3.16 mg/dL prior to discharge. During hospitalization, nonsteroidal anti-inflammatory drugs and antihypertensive medications were administered and continued after discharge. The patient was discharged in a stable condition on postoperative Day 13.

**Figure 3:**
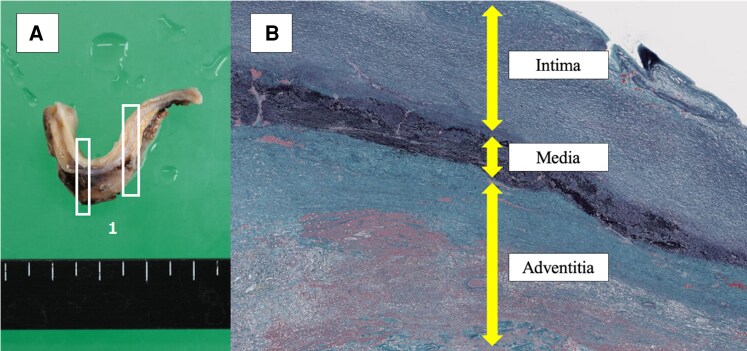
Histopathological findings of the resected aortic wall (Elastica-Masson stain). (*A*) Macroscopic view showing diffuse thickening of the aortic wall. (*B*) Microscopic view showing hyaline degeneration and inflammatory cell infiltration in the adventitia, alongside occlusion of the vasa vasorum, haemorrhage, and congestion. The media shows loss of elastic fibres and structural disorganization, and the intima exhibits mild thickening.

The histopathological diagnosis of TA was confirmed at the time of discharge, and the patient was promptly referred to a rheumatology department at another hospital for specialized management. However, he returned to our hospital before this scheduled appointment, on postoperative Day 19 (Day 6 after discharge), with a persistent low-grade fever, fatigue, and back pain. Laboratory testing revealed a substantially elevated CRP level of 20.52 mg/dL, consistent with ongoing systemic inflammation.

Contrast-enhanced CT revealed a new anastomotic pseudoaneurysm between the left common carotid artery and the prosthetic graft (*Figure 4*). Emergency endovascular repair performed via a retrograde approach through the left common carotid artery using a 10 × 50-mm VIABAHN stent graft (W. L. Gore & Associates, Flagstaff, AZ, USA) successfully sealed the contrast leak (*Figure 5*). High-dose intravenous methylprednisolone (1000 mg/day) was initiated the day after the procedure and continued for 3 days, followed by oral prednisolone (1 mg/kg/day). The clinical course was favourable, and the patient was re-referred and transferred to the rheumatology department on postoperative Day 15 following the second intervention.

**Figure 4:**
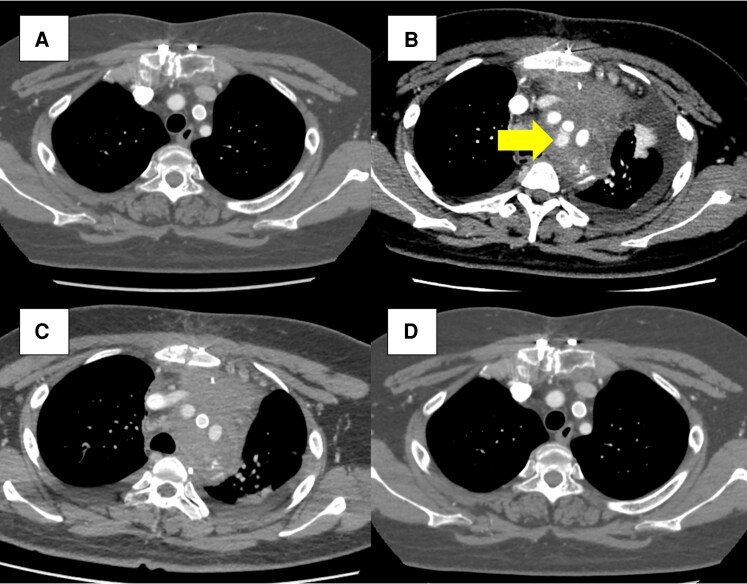
Serial contrast-enhanced computed tomography images of an anastomotic pseudoaneurysm between the left common carotid artery and the prosthetic graft. (*A*) Immediate postoperative image showing no pseudoaneurysm. (*B*) Image obtained at readmission demonstrating an anastomotic pseudoaneurysm (arrow). (*C*) Image after endovascular stent grafting showing resolution of the pseudoaneurysm. (*D*) Six-month follow-up image showing no recurrence.

**Figure 5:**
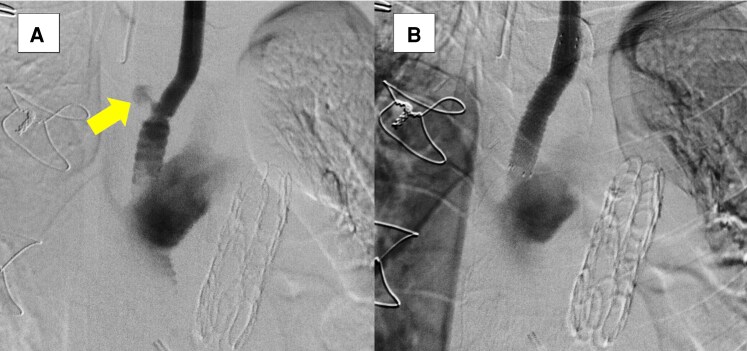
Angiographic findings during the endovascular stent grafting. (*A*) Intraoperative angiography showing contrast extravasation from the anastomotic site between the left common carotid artery and the prosthetic graft (arrow). (*B*) Complete resolution of the contrast leak following deployment of a 10 × 50-mm VIABAHN stent graft.

After transfer, steroids were tapered, and additional immunosuppressive therapy with methotrexate and infliximab was initiated, resulting in remission. Follow-up PET-CT performed approximately 3 months after the initial surgery showed no abnormal uptake suggestive of active vasculitis. At the 6-month follow-up, contrast-enhanced CT confirmed that the anastomotic site remained intact without recurrence or complications. Ongoing management includes regular monitoring of inflammatory markers and periodic imaging follow-up.

## Discussion

We described the case of a 20-year-old man with a giant ascending aortic pseudoaneurysm, which is atypical for TA in terms of aneurysm type, anatomical location, and patient demographics.

In younger patients presenting with unexplained systemic inflammation and aortic pseudoaneurysm, differential diagnoses should include not only TA but also vasculo-Behçet’s disease, infectious aortitis, and syphilitic aortitis. Notably, pseudoaneurysms account for approximately 50% of all arterial lesions in Behçet’s disease, which predominantly affects young men.^7^ This case highlights the importance of considering atypical vascular presentations in younger patients and broadens the clinical spectrum of TA.

Although pulmonary artery compression caused by anastomotic pseudoaneurysms following aortic surgery has been reported previously,^8,9^ a native aortic pseudoaneurysm leading to such compression and subsequent acute right heart failure is exceedingly rare. Pseudoaneurysmal pulmonary compression resulted in right ventricular dilatation, tricuspid regurgitation, and inferior vena cava enlargement in our patient, ultimately culminating in a pre-shock state. Rapidly expanding pseudoaneurysms can abruptly obstruct pulmonary arterial flow, potentially causing acute circulatory collapse if not promptly treated.

Anastomotic pseudoaneurysm is a severe postoperative complication in patients with TA. A 20-year follow-up study reported a cumulative incidence of 13.8%, highlighting the importance of long-term vascular surveillance.^10^

Preoperative control of vascular inflammation is essential to reducing postoperative risk.^11,12^ When possible, surgical intervention should be deferred until disease activity has subsided.^13^ Glucocorticoids and immunosuppressive agents are commonly used to achieve inflammation control.^13^

In the present case, emergency surgery was prioritized because of the patient’s critical clinical condition, and the diagnosis of TA was made only after a postoperative histopathological examination. The initiation of immunosuppressive therapy was therefore delayed, which may have contributed to the development of the anastomotic pseudoaneurysm; however, this association remains suggestive rather than clearly demonstrated. Furthermore, the optimal management of TA often requires comprehensive care, including medical therapy, and close collaboration with other centres may facilitate appropriate treatment.

Although surgical options for anastomotic pseudoaneurysm, such as direct anastomosis, patch repair, and graft replacement have been reported,^14^ these procedures are technically challenging to perform during the early postoperative period, particularly when inflammation is ongoing and adhesions are severe. In such situations, endovascular stent grafting may serve as a safer and less invasive alternative.

In the present case, retrograde access via the left common carotid artery facilitated accurate stent deployment with minimal manipulation of the anastomotic site, thereby reducing the risk of further pseudoaneurysm formation.

The follow-up period was relatively short, limiting assessment of long-term vascular outcomes and durability of the intervention.

This case highlights a rare presentation of Takayasu arteritis with an ascending aortic pseudoaneurysm. It also underscores the importance of treatment strategies for postoperative anastomotic pseudoaneurysm and perioperative inflammation control.

## Data Availability

The data underlying this article are available from the corresponding author upon reasonable request.

## Lead author biography

Dr. Shota Inoue graduated from St. Marianna University School of Medicine in 2020 and completed both his initial and advanced surgical training at Toranomon Hospital. He has served as a cardiovascular surgeon at Sendai Kosei Hospital since his sixth year of clinical practice.

## Notes

**Consent:** Written informed consent was obtained from the patient for the publication of this case.
